# Electron crystallography with the EIGER detector

**DOI:** 10.1107/S2052252518000945

**Published:** 2018-02-14

**Authors:** Gemma Tinti, Erik Fröjdh, Eric van Genderen, Tim Gruene, Bernd Schmitt, D. A. Matthijs de Winter, Bert M. Weckhuysen, Jan Pieter Abrahams

**Affiliations:** a Swiss Light Source Detector Group, Paul Scherrer Institute, Villigen, Switzerland; b Laboratory of Biomolecular Research, Paul Scherrer Institute, Villigen, Switzerland; c Inorganic Chemistry and Catalysis, Debye Institute for Nanomaterials Science, Utrecht, The Netherlands; d Center for Cellular Imaging and NanoAnalytics, University of Basel, Basel, Switzerland

**Keywords:** EIGER, hybrid pixel detectors, electron crystallography, SAPO-34

## Abstract

Electron crystallography is a discipline that currently attracts much attention as method for inorganic, organic and macromolecular structure solution. EIGER, a direct-detection hybrid pixel detector developed at the Paul Scherrer Institut, Switzerland, has been tested for electron diffraction in a transmission electron microscope. EIGER features a pixel pitch of 75 × 75 µm^2^, frame rates up to 23 kHz and a dead time between frames as low as 3 µs. Cluster size and modulation transfer functions of the detector at 100, 200 and 300 keV electron energies are reported and the data quality is demonstrated by structure determination of a SAPO-34 zeotype from electron diffraction data.

## Abstract 2

An EIGER detector with 256 × 256 pixels has been used to collect electron diffraction data from a SAPO-34 crystal. The data quality from the EIGER detector is comparable with other hybrid pixel detectors available for electron diffraction.

## 1. Introduction   

Crystallography is an important technique for solving the molecular structures of inorganic, organic and macromolecular compounds. In organic and macromolecular crystallography, where the rotation method has been routine for data aquisition, hybrid pixel detectors have enabled shutterless data collection (Pflugrath, 1999 ▸). In electron diffraction, the rotation method was pioneered by Kolb *et al.* (2011 ▸) and lately the first results were presented from shutterless data collection. Two types of direct electron detector (McMullan *et al.*, 2016 ▸) are now in use for transmission electron microscopes (TEMs): (i) hybrid pixel detectors for diffraction studies and (ii) monolithic active pixel sensors (MAPSs) for imaging applications. Both are fabricated with standard complementary metal oxide semiconductor (CMOS) technology. Hybrid pixel detectors require an additional per-pixel bump bonding of the front-end electronics to a sensor layer.

MAPS detectors are more suited to higher electron energies due to their reduced thickness, which minimizes electron scattering within the sensor (McMullan *et al.*, 2014 ▸, 2009 ▸). The Medipix family of hybrid pixel detectors (Llopart *et al.*, 2002 ▸; Ballabriga *et al.*, 2011 ▸) have previously shown good spatial resolution between 60–120 keV electron energies (McMullan *et al.*, 2007 ▸, 2009 ▸; Mir *et al.*, 2017 ▸). However, hybrid pixel detectors are currently not suited to high-resolution imaging of electrons with energies above 100 keV due to their large point spread, but, unlike monolithic detectors, hybrid pixel detectors are ideally suited for measuring electron diffraction patterns due to their high dynamic range, on-pixel electron-counting capabilities, radiation hardness and high frame rate (Nederlof *et al.*, 2013 ▸; van Genderen *et al.*, 2016 ▸). Proof-of-principle measurements using high frame rate and high dynamic range hybrid pixel detectors to record diffraction patterns and perform ptychography experiments on scanning TEMs have also recently been shown (Krajnak *et al.*, 2016 ▸; Tate *et al.*, 2016 ▸).

The EIGER hybrid pixel detector (Dinapoli *et al.*, 2011 ▸), developed at the Paul Scherrer Institut (PSI), Switzerland, is primarily aimed at photon detection in diffraction experiments at synchrotrons, including applications in protein crystallography (Casanas *et al.*, 2016 ▸), coherent diffraction imaging and ptychography (Guizar-Sicairos *et al.*, 2014 ▸), X-ray photon correlation spectroscopy (Johnson *et al.*, 2012 ▸) and wide-angle X-ray scattering experiments. EIGER is also suitable for low-energy (8–20 keV) electron detection in photo-emission electron microscopy experiments (Tinti *et al.*, 2017 ▸).

In this paper we report on the use of EIGER in a TEM at 100, 200 and 300 keV electron energies. Section 2 describes the experimental setup, and Sections 3 and 4 present the detector concept and characterization and calibration for electron detection in the studied energy range. Simulations in Section 5 validate the spatial resolution results. As a proof of concept, we measured electron diffraction data from thinned synthetic silicoaluminophosphate (SAPO-34) crystals (Lok *et al.*, 1984 ▸) and solved the structure by direct methods. These results are presented in Section 6. SAPO-34 is of academic and industrial interest for its excellent properties as a heat adsorbent (Fischer, 2015 ▸) for gas purification (Carreon *et al.*, 2008 ▸) and as a heterogeneous catalyst, in particular for methanol-to-olefin (MTO) reactions (Stöcker, 1999 ▸; Vora *et al.*, 2009 ▸). Large microcrystals of SAPO-34 (50 × 50 × 50 µm^3^) were described previously (Mores *et al.*, 2008 ▸; Qian *et al.*, 2013 ▸; Karwacki *et al.*, 2007 ▸).

## 2. Experimental setup   

### 2.1. The Polara microscope   

The experiments were performed on a Polara 300 kV microscope (FEI, Eindhoven, The Netherlands) at the Center for Cellular Imaging and NanoAnalytics (C-CINA), Basel. The microscope is capable of operating at acceleration voltages of 100–300 kV. Flat-field and knife-edge experiments were performed at 4.7 k magnification with a 70 µm C2 aperture. General alignment procedures were followed for 100, 200 and 300 kV, respectively. The knife-edge images were created by the shadow of the beam block. The dose was adjusted by converging and diverging the beam. For the rotation/diffraction experiments, the goniometer rotation speed was set with the Polara *TEMspy* software and a 10 µm C2 aperture was used to create an almost-parallel beam of 2 µm diameter on the specimen. The EIGER detector was mounted on-axis, using an interface flange between the microscope and camera as described by van Genderen *et al.* (2016 ▸) and an adapted Pb shielding of an in-house developed Timepix camera (Llopart *et al.*, 2007 ▸).

### 2.2. Detector mounting   

**Figure 1:**
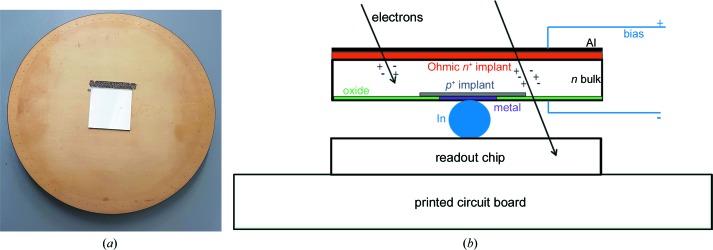
(*a*) Photograph of the PCB supporting the detector with a single (2 × 2 cm^2^) chip. (*b*) A schematic representation of the layers of a hybrid pixel detector.

The sensitive area of the detector used in this experiment was ∼2 × 2 cm^2^. The detector was a single chip assembly glued onto a printed circuit board (PCB), as shown in Fig. 1 ▸(*a*). The gold-coated PCB acted as a vacuum barrier, with 50 cm long cables connecting the back of the PCB to the readout board, passing through the Pb shielding cylinder of the Polara microscope. The readout board sat outside of the shielding and was water-cooled to dissipate the heat from the field-programmable gate arrays (FPGAs), while no active cooling was applied to the detector as the static power consumption is only 0.83 W per chip.

### 2.3. Synthesis of SAPO-34 crystals   

The synthesis procedure for the large SAPO-34 crystals is described by Karwacki *et al.* (2007 ▸). As the crystals themselves are too large to be imaged by the TEM, thin electron-transparent sections were made using a focused ion beam scanning electron microscope (FIB-SEM) (Nova Nanolab 600 Dualbeam, FEI) equipped with an Omniprobe micromanipulator (Oxford Instruments, Abingdon, UK) following standard procedures (Karwacki *et al.*, 2009 ▸). The thickness of the sample was 150 nm.

## 3. The EIGER detector   

EIGER is a hybrid pixel detector consisting of a sensor bonded to an application-specific integrated circuit (ASIC), which processes the signals coming from the sensor. Each pixel in the sensor is connected to the corresponding pixel in the ASIC: the pixel matrix consists of 256 × 256 pixels, each with a size of 75 × 75 µm^2^. The ASIC is designed in UMC 0.25 µm technology and the sensor used in the experiment is a 320 µm thick Si layer manufactured by Hamamatsu (Hamamatsu City, Japan). The entrance window, *i.e.* the first part of the sensor seen by the radiation, is composed of ∼1 µm thick Al, enabling a bias voltage to be applied to the sensor, and an *n* ^+^ implant ∼2 µm thick. The entrance window does not detect the signal coming from the radiation. A schematic diagram of the detector concept (sensor and readout ASIC) is shown in Fig. 1 ▸(*b*); more details can be found in the work of Johnson *et al.* (2014 ▸) and Tinti *et al.* (2017 ▸).

The primary electrons from the TEM enter the sensor and interact with the electrons of the Si: primary electrons lose energy following the Bethe–Block equation and the ionization maximum happens at the end of their tracks. Along the electron path, electron–hole pairs are produced. With the applied bias voltage (150 V in the experiments presented here), holes drift towards the pixelated part of the sensor, where the charge is collected. There are two effects that contribute to detection of the signal in multiple neighbouring pixels: (i) the charge generated within the sensor diffuses laterally during the drift towards the electrode; (ii) the primary electrons scatter elastically, changing the direction of their path. Due to the latter effect, the electron track can cover several pixels.

From simulations, electrons with energies below 200 keV do not reach the ASIC and no radiation damage is expected for the Si sensor either: the electrons do not penetrate deeply enough to reach the pixelated side of the sensor, so they cannot build up positive charge in the oxide (visible in Fig. 1 ▸ *b*). So up to those energies, there is no increase in the sensor leakage current due to surface radiation damage. Only electrons above 260 keV (Rossi *et al.*, 2006 ▸) have sufficient energy to create bulk damage in the Si sensor. However, 300 keV electrons can go through the 320 µm thick sensor and reach the ASIC. So for this energy, the ASIC radiation hardness needs to be considered, in addition to the sensor radiation damage (both surface and bulk). EIGER has been designed with radiation hardening techniques (Annelli, 2000 ▸) and it is expected to be radiation hard up to 0.3 kGy.

The signal in the ASIC is first amplified with a user-configurable gain and shaped. The signal is compared with a programmable threshold and if the pulse amplitude is higher than the threshold, a counter in the pixel is incremented. The counter can be configured in 4-, 8- or 12-bit mode. The threshold is set globally for the ASIC, but 6-bit pixel-specific shifts can be applied to the threshold to minimize the pixel-to-pixel differences. The readout board is able to sum images extending the dynamic range from 12-bit to 32-bit and can also store images in memory. The data are transferred from the readout board through a 1 or 10 GbE connector. By latching the counter values onto capacitors, EIGER is able to acquire an image while the previous one is being read out, operating with a minimum dead time of 3 µs for 12-bit images. The maximum frame rates are 23, 12 or 6 kHz in 4-, 8- or 12-bit mode, respectively, and 2 kHz in 32-bit mode.

At high particle rates, pile-up effects induce a deviation from linearity between counts and the incoming flux. The deviation is dependent on the preamplifier gain settings. For the settings used in these measurements, the count rate capability was measured with photons to deviate 10% in linearity for more than 0.7 Mcounts pixel^−1^ s^−1^ or 126 Mcounts mm^−2^ s^−1^. In the measurements presented in Section 6, the rates per pixel were at least an order of magnitude lower to avoid deviation from linearity.

## 4. Characterization of the EIGER detector for TEM   

### 4.1. Calibration with X-rays   

**Figure 2:**
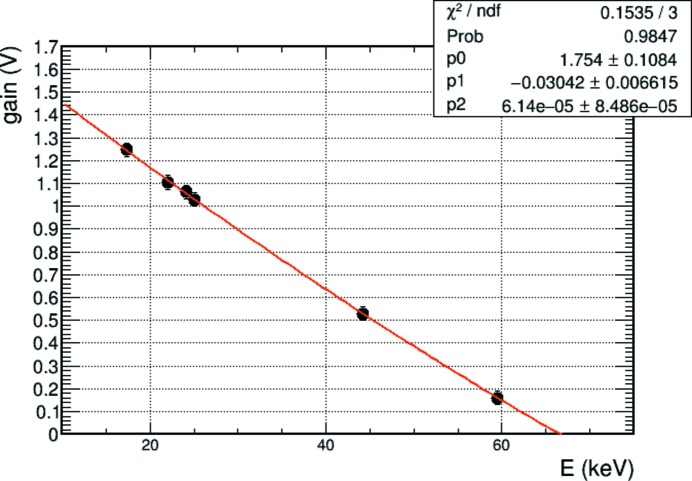
Gain *versus* energy calibration curves. The intercept with the horizontal axis indicates that the maximum threshold that can be set is at ∼67 keV.

Unlike electrons, photons deposit all their energy at once. Therefore, energy calibration of the detector can be performed *versus* absolute energy using X-ray fluorescence photons. Traditionally, the pre-amplifier gain is kept constant for a range of energies and the threshold voltage is calibrated *versus* energy (Tinti *et al.*, 2015 ▸). Alternatively, the threshold can be fixed and the preamplifier gain can be calibrated *versus* energy (Johnson *et al.*, 2014 ▸). The latter method has the advantage in EIGER of covering a larger range of energies by changing only one parameter and was therefore chosen for the present work. A high-gain shaper setting was selected to minimise the noise. Gain scans were performed at each fluorescence energy and the inflection point parameter (the turning point of the scan curve) was extracted through analytical modelling of the scan curve (Johnson *et al.*, 2014 ▸). Fig. 2 ▸ shows the gain *versus* energy calibration obtained using Mo, Ag, In, Sn and Tb fluorescence targets. A final calibration point, at ∼60 keV, was collected using an ^241^Am source. A maximum threshold of ∼67 keV can be reached. The value of the gain needed to distinguish the signal is obtained by gain = *p*_0_ + *p*_1_ *E* + *p*_2_ *E* ^2^, where *E* is the threshold energy in kiloelectronvolts and *p*_0_, *p*_1_ and *p*_2_ are the coefficients determined from the gain *versus* energy curve in Fig. 2 ▸. Pixel-to-pixel corrections to equalize the threshold within the chip were also performed using an Sn fluorescence target and they were applied for the whole energy range covered.

### 4.2. Measurements with electrons   

#### 4.2.1. Cluster size analysis   

**Figure 3:**
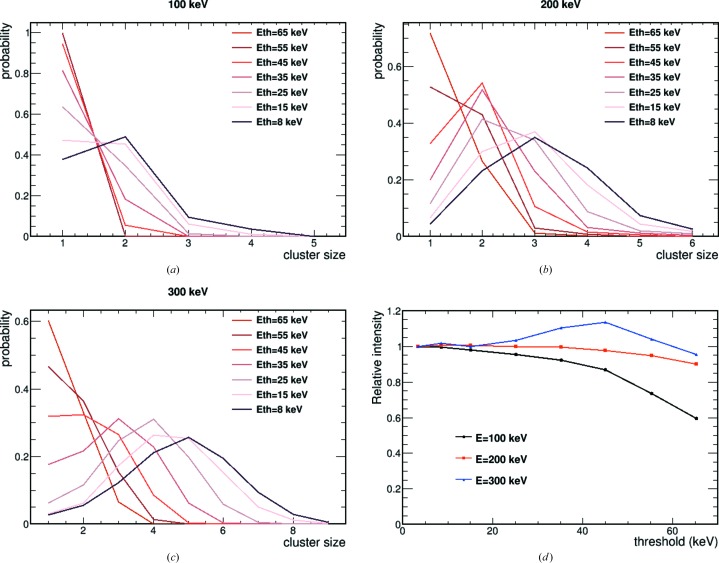
(*a*), (*b*) and (*c*) Cluster size as a function of threshold setting (*E*_th_) for 100, 200 and 300 keV electrons, respectively. The distributions have been normalized such that their integral is unity. (*d*) The relative number of detected clusters as a function of threshold for the three different electron energies. The data at the various thresholds have been normalised to the lowest measured threshold data.

The microscope intensity was lowered to detect single electrons. The size in pixels of single-electron interactions was studied as a function of the threshold at 100, 200 and 300 keV electron energies. A cluster was defined as neighbouring pixels above threshold, with an eight-pixel connectivity algorithm. Fig. 3 ▸(*a*) shows the cluster size for 100 keV electrons for thresholds between 8 and 65 keV. Above 45 keV, only single-pixel clusters are detected (at 95%), as expected if the electron interaction is contained within one pixel but two pixels collect charge from the same primary electron interaction due to diffusion effects. Figs. 3 ▸(*b*) and 3 ▸(*c*) show the equivalent cluster size distribution as a function of threshold setting for 200 and 300 keV, respectively. At these high energies, the same electron undergoes a random walk due to multiple scattering along its track, depositing energy in multiple pixels. When setting a low threshold (8 keV) for the readout chip, the same electron is detected in multiple neighbouring pixels (respectively peaking at 3 and 5 pixels per cluster): multiple-pixel clusters are due to multiple scattering. Increasing the threshold to 65 keV results in mostly single-pixel clusters. However, selecting a single pixel above a high threshold does not correspond to selecting the pixel at which the electron entered the sensor. Fig 3 ▸(*d*) shows the relative intensity of counted electrons as a function of threshold: by increasing the threshold the efficiency in detecting electrons is expected to decrease. This happens for 100 keV electrons, where a high threshold allows single-electron hit selection. For 200 and 300 keV electrons, though, by increasing the threshold up to 65 keV, single-hit clusters are recorded in the majority but the total number of clusters (of any size) remains almost unchanged from 10–65 keV. This means that by increasing the threshold we record the most energetic hit per cluster, but in reality the electrons enter the detector sensor in another pixel. Therefore, the spatial resolution could be worse for single-pixel clusters than for larger clusters. For 300 keV, at a threshold of around 40 keV, one apparently detects more events. This is actually due to the fact that, at those threshold settings, a single-electron event could be split into multiple unconnected clusters. A more advanced clustering algorithm, however, might be able to merge the split events.

#### 4.2.2. Modulation transfer functions   

In order to study the spatial resolution, we used the shadow of the beam-stop of the microscope as a straight edge. The edge was oriented at a small angle to the pixel matrix. Flat-field images were also collected and were used to correct the knife-edge images before analysing them. The edge data and the flat-field correction were taken at multiple threshold energies and at different electron energies. For each row of the pixel matrix, the edge tilting angle was corrected for to allow sub-pixel sampling. The normalized intensity distribution *n*(*x*) was fitted as follows:
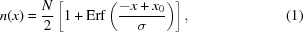
where σ is the fitted resolution and *N* is the amplitude normalization, and the shift to the edge centre *x*_0_ = 0 if the correction of the tilting angle is correctly applied.

**Figure 4:**
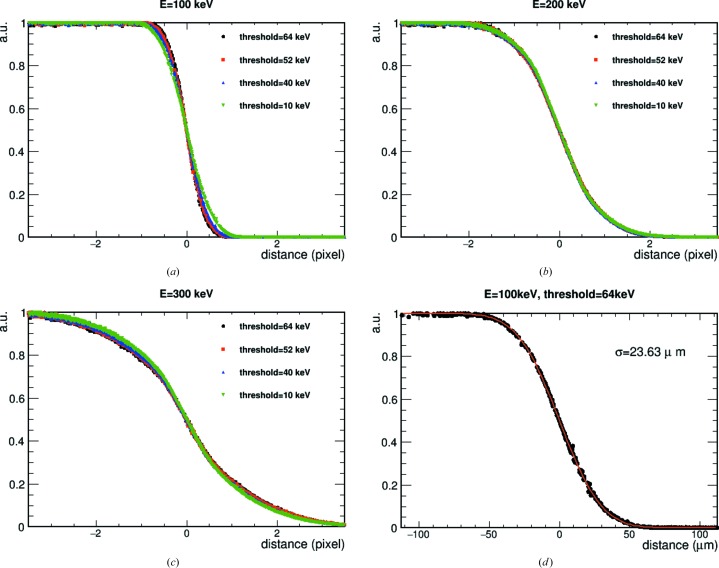
The edge spread function for different threshold settings for (*a*) 100, (*b*) 200 and (*c*) 300 keV electrons. (*d*) The edge spread function and corresponding fit for the best resolution case, obtained for 100 keV electrons and threshold set at 64 keV.

Figs. 4 ▸(*a*), 4 ▸(*b*) and 4 ▸(*c*) show the edge results for different threshold energies of 100, 200 and 300 keV electrons, respectively. Increasing the threshold improves the spatial resolution for 100 keV electrons: the fitted resolution value, obtained from equation (1), goes from 33 µm at 10 keV threshold to 24 µm at 64 keV threshold, as shown in Fig. 4 ▸(*d*). The expected ideal resolution for a single EIGER pixel is σ_i_ = 21.65 µm, obtained as the pixel size divided by 12^1/2^. However, increasing the threshold does not improve the spatial resolution for the 200 and 300 keV electron data. For 200 keV electrons, the best measured resolution is 57 µm at 10 keV threshold, while it worsens slightly to 58 µm at 64 keV threshold. For the 300 keV data, the best resolution is 91 µm at 10 keV threshold and this is lowered to 101 µm for a threshold setting at 64 keV. The edge spread function data are consistent with the expectations from cluster size analysis. For the 200 and 300 keV data, using a 10 keV threshold gives a better spatial resolution than that obtained for high thresholds, as by detecting the whole cluster the pixel corresponding to the electron entrance into the sensor is included, which is instead excluded when the threshold is raised.

**Figure 5:**
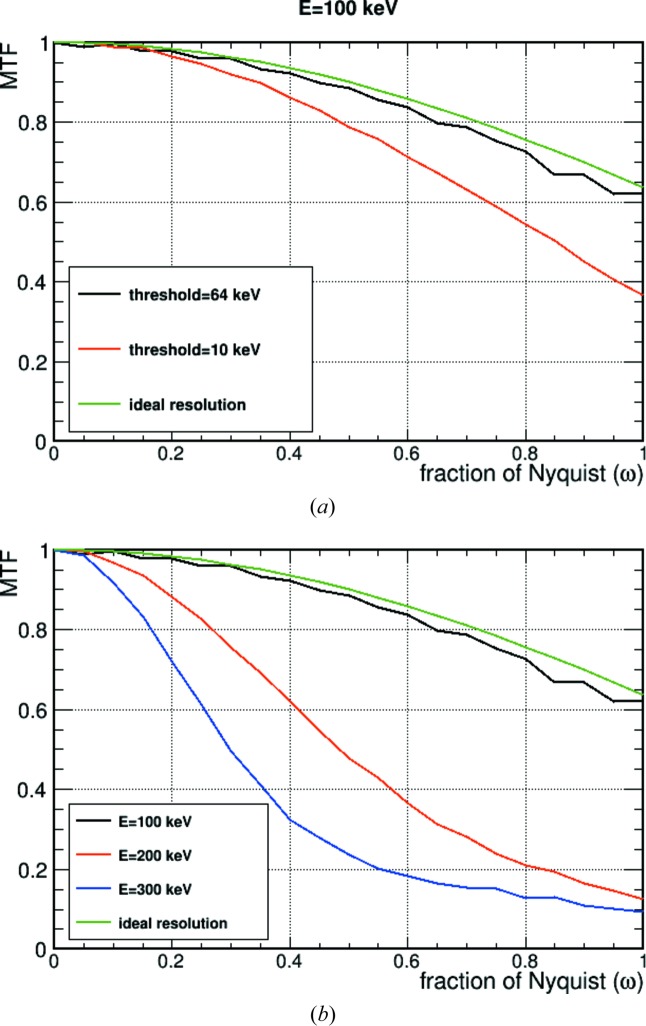
(*a*) MTF as a function of Nyquist frequency for 100 keV electrons. Two different threshold settings and the ideal MTF are plotted. (*b*) MTF as a function of Nyquist frequency for 100, 200 and 300 keV electrons. The best MTF at 100 keV is plotted, *i.e.* setting the threshold at 64 keV. For 200 and 300 keV electrons, the threshold is set to 10 keV.

Based on the edge data, we studied the modulation transfer function (MTF), *i.e.* the variation in response of a detector to a sinusoidal input signal. The edge spread function is differentiated with respect to the pixel unit coordinate to obtain the line spread function. The Fourier transform of the line spread function results in the MTF. Fig. 5 ▸(*a*) shows the MTFs as a function of Nyquist frequency (ω) for different threshold settings for 100 keV electrons and the ideal MTF for a pixel detector, which is calculated as follows (McMullan *et al.*, 2009 ▸):
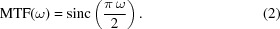
One can clearly see the effect of changing the threshold for 100 keV electrons: the highest possible threshold of 64 keV makes the detector very close to an ideal one. Fig. 5 ▸(*b*) shows the MTF as a function of Nyquist frequency for 100, 200 and 300 keV electrons. For the 100 keV electrons, the threshold setting which gives the best resolution (*i.e.* the 64 keV threshold) is plotted. At this electron energy, the MTF of EIGER is close to that of an ideal pixel detector of 75 × 75 µm^2^ pixel pitch. EIGER can be used for both electron imaging and diffraction. For the 200 and 300 keV data, only the threshold setting at 10 keV is plotted, as it gives the best spatial resolution for those energies for the available threshold range. At 200 and 300 keV, the MTFs are very far from that of an ideal detector, making EIGER less suitable for imaging experiments, while its high frame rate capability still makes it interesting for electron diffraction detection even at higher electron energies.

## 5. Simulations   

To better understand the performance of the EIGER detector and the possibilities of using hybrid pixel detectors for electron diffraction, the measurement setup used in the edge experiment was simulated in *Geant4* (Agostinelli *et al.*, 2003 ▸) using the *Geant4*–Medipix framework (Schübel *et al.*, 2014 ▸). The *emstandard_opt3* physics list constructor was used and, after energy deposition, a charge-transport model with drift/diffusion, but neither a pixel weighting field nor a preamplifier model was used. The per pixel energy deposition from each event was stored in a binary file and the threshold was applied in a second step, allowing for multiple thresholds without rerunning the full simulation.

The simulated sensor was 320 µm thick Si with 75 × 75 µm^2^ pixels, as in the EIGER sensor used in the experiment, but a reduced area of 51 × 51 pixels was simulated. Indium bump bonds with 10 µm radii were placed between the sensor and an Si readout chip to take backscattering from layers behind the sensor into account, even though only 300 keV electrons go through the sensor. As an edge, 300 µm thick tungsten tilted at 5° was used. To avoid edge effects, ten rows on the top and bottom were discarded, as were the ten outer columns on each side. The sensor was uniformly illuminated with electrons impinging perpendicularly onto the sensor surface. The oversampled edge was constructed from the simulated image. By simulating the full image instead of a single point we have the possibility of making a direct comparison between the measured data and our simulations.

**Figure 6:**
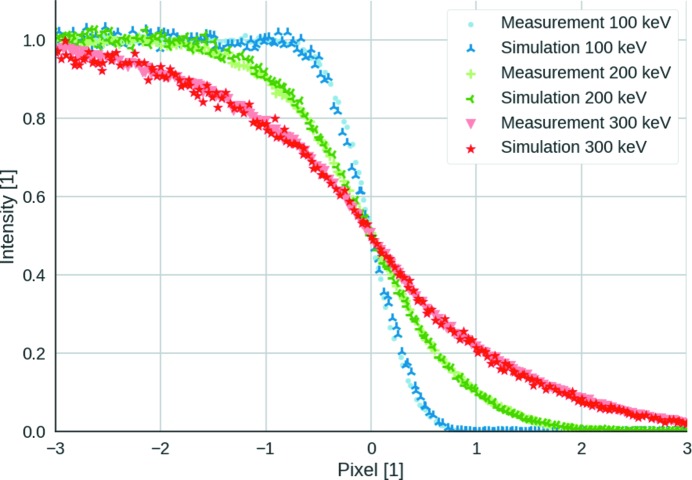
Comparison between the measured and simulated edge for 60 keV threshold.

**Figure 7:**
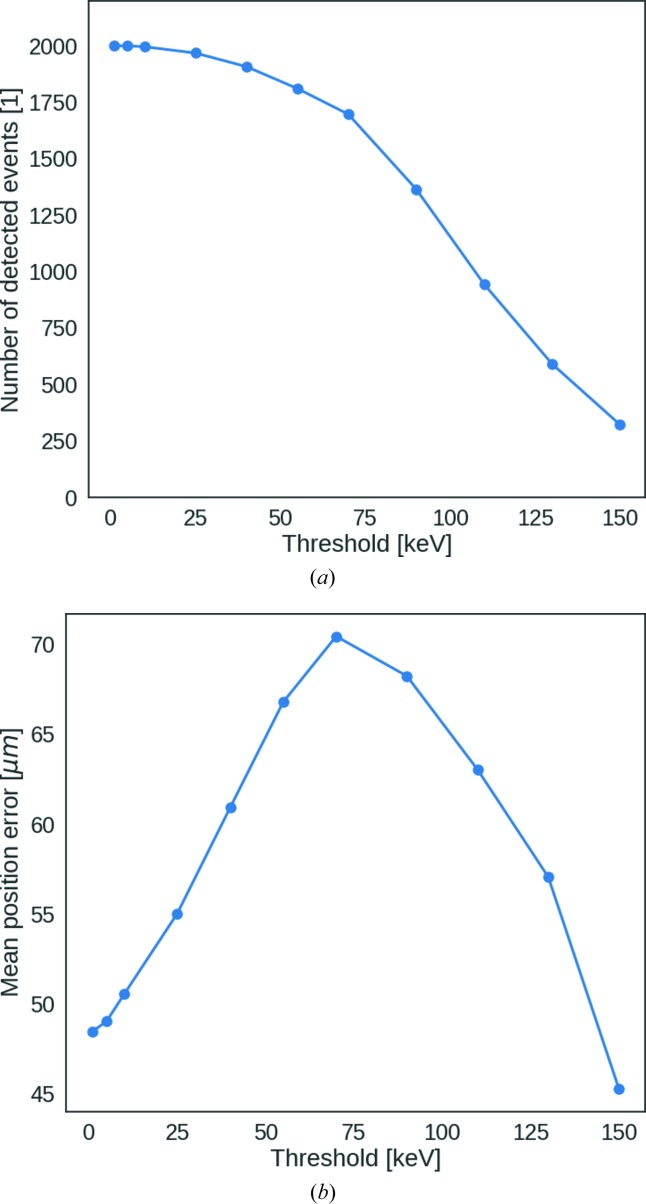
(*a*) Simulation of the number of detected events as a function of threshold for 200 keV electrons. (*b*) Simulation of the mean position error as a function of threshold for 200 keV electrons.

For 100, 200 and 300 keV there is good agreement between the measurements and simulations, as shown in Fig. 6 ▸ for a threshold of 60 keV. The trends in the simulations agree with the measurements, allowing the prediction of the edge results from threshold settings higher than 65 keV, which were not measured during this beamtime. The experimental data showed that, for the 200 and 300 keV electrons, the edge became less sharp with higher thresholds. This effect is also shown by the simulations. The relative loss of intensity due to the threshold reduction is plotted in Fig. 7 ▸(*a*). Fig. 7 ▸(*b*) shows the mean position error, defined as the distance between the centroid of the cluster and the point where the electron enters the sensor. By increasing the threshold up to 65 keV, we lose information on the entrance point of the electron. An improvement in the mean position error is only possible by further increasing the threshold, which compromises the efficiency, as only those electrons that do not scatter outside the entrance pixel are observed in that case. While theoretically the maximum value of the threshold of EIGER can be raised by altering the shaper setting, this has not been attempted as it would not allow better detector performance for 200 or 300 keV electrons.

With such a good agreement between simulations and data, we used the simulation framework of the edge response as described above to extract the MTF function for 120 keV electrons. We found an MTF value of 0.48 at the Nyquist frequency for a threshold setting of 65 keV. When comparing the MTF results from EIGER at 120 keV electron energy with current state-of-the-art electron detectors, we conclude that EIGER has an MTF which is greater than those from the Film SO-163, the CCD TVIPS 224 and the Medipix2 detectors at the Nyquist frequency, as reported in Fig. 5 of McMullan *et al.* (2009 ▸). Compared with the Medipix2 detector, which is another hybrid pixel detector with a 55 µm pixel pitch, the value of the MTF as extracted from the plot in the work by McMullan *et al.* (2009 ▸) at the fraction of the Nyquist frequency for the EIGER pixel pitch (55/75 = 0.73) is approximately 0.5, consistent with the EIGER findings. However, due to the multiple scattering of the electrons interacting in the Si sensor, reducing the pixel size to values smaller than 75 µm would not improve the MTF for hybrid pixel detectors sensitive to electrons in the energy range 100–300 keV.

## 6. Structure reconstruction of a zeotype   

Diffraction data were collected at room temperature from SAPO-34, a zeotype with a CHA framework (http://europe.iza-structure.org/IZA-SC/framework.php?STC=CHA). The electron energy was 200 keV, corresponding to an electron wavelength of λ = 0.02508 Å. Two different threshold levels were used, 25 keV and 60 keV. Data were collected according to the rotation method (Arndt & Wonacott, 1977 ▸; Zhang *et al.*, 2010 ▸; Kolb *et al.*, 2011 ▸; Yun *et al.*, 2015 ▸) and processed with *XDS* (Kabsch, 2010 ▸) with the Laue group 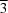 and nominal cell parameters. The following parameters were refined during data processing: virtual detector distance and orientation, beam direction, and rotation axis. The virtual detector distance was estimated from an Al powder pattern. The rotation axis was estimated by eye from reflections that stay longest in the diffraction condition. The unit-cell parameters were determined by indexing. The oscillation width was calculated as ΔΦ/(No. of frames) and refined by minimising the deviation of the cell parameters from a rhombohedral lattice. In order to get estimates for the cell uncertainties, the cell was refined and the detector distance was kept constant as a last step in *XDS*. Data statistics are shown in Table 1 ▸.

**Figure 8:**
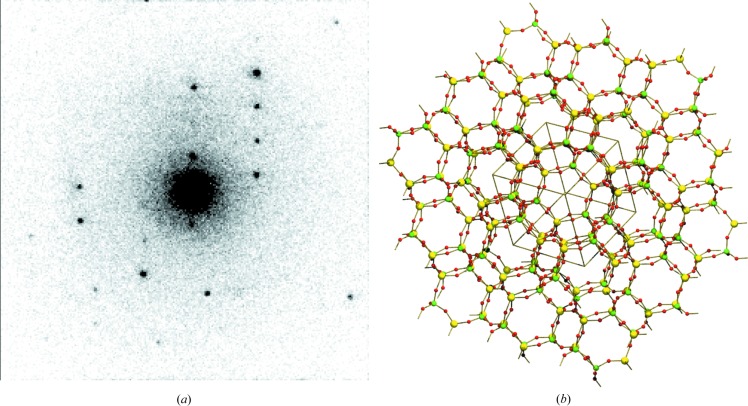
(*a*) A sample electron diffraction pattern for SAPO-34. (*b*) The structure of the SAPO-34 framework with *T* sites (P and Al, shown as green and yellow spheres, respectively) and oxygen atoms (shown as small red spheres).

The structure was solved with *SHELXT* (Sheldrick, 2015*b* ▸), refined with *SHELXL* (Sheldrick, 2015*a* ▸) and modelled with *SHELXLE* (Hübschle *et al.*, 2011 ▸). *SHELXT* was set to search for aluminium, phosphorus and oxygen in Laue group 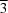. In both data sets, both *T* sites and the four O sites were correctly assigned by *SHELXT*. No attempt was made to model the template. Model statistics are shown in Table 2 ▸. Note that the detector distance was recalibrated against the target P—O distance of 1.581 Å for the data set with threshold 25 keV, *i.e.* its Al—O bond distance, and both the P—O and Al—O bond distances for the data set with threshold 60 keV, listed in Table 3 ▸, act as validation for the data quality. A diffraction pattern recorded on the detector is shown in Fig. 8 ▸(*a*) and the framework structure is shown in Fig. 8 ▸(*b*).

## 7. Conclusions   

We have used the EIGER hybrid pixel detector to determine the structure of a SAPO-34 zeotype on the Polara electron microscope at C-CINA, Basel. The detector was characterized with electron energies of 100, 200 and 300 keV. The MTF of EIGER was found to be close to that of an ideal detector with 75 × 75 µm^2^ pixels for electron energies in the 100 keV range. By increasing the threshold settings above 65 keV, one obtains single-pixel clusters for 100 keV electrons and a spatial resolution of ∼24 µm. The MTF value in this case is higher than 0.62 at the Nyquist frequency. The spatial resolution is not as good as the ideal value for 200 and 300 keV electrons as the electron multiple scattering within the Si sensor results in many pixel hits. It is not possible to restore single-pixel resolution even by increasing the threshold, as the pixel where the most energy is deposited is far from the electron entrance point, resulting in a worse spatial resolution at higher threshold values. The MTFs for 200 and 300 keV electrons have values lower than 0.15 at the Nyquist frequency. In addition, for 300 keV electrons one has to take into consideration radiation hardness problems causing bulk and surface damage of the Si sensor and the radiation hardness of the ASIC, as 300 keV electrons are not stopped by the Si sensor. Nevertheless, we have demonstrated the high quality of the EIGER detector by reconstructing a SAPO-34 zeotype structure in an experiment with 200 keV electrons.

We foresee an opportunity to use the EIGER pixel detector in electron crystallography and microscopy up to 200 keV electron energy, in particular thanks to its extremely high frame rate (up to 23 kHz in 4-bit mode). This would allow looking at single-electron events and would reduce the effect of the drift of the sample in short exposures. We are in the process of producing a larger detector (2 × 2 readout chips bonded to a single sensor of area 4 × 4 cm^2^). Even larger detectors could be produced, although with a dead area. Large detectors are appealing to improve the MTF while retaining high efficiency, when combined with an increase in (virtual) camera distance.

## Supplementary Material

Crystal structure: contains datablock(s) I. DOI: 10.1107/S2052252518000945/sp5002sup1.cif

Structure factors: contains datablock(s) I. DOI: 10.1107/S2052252518000945/sp5002Isup2.hkl

CCDC reference: 1817054

## Figures and Tables

**Table 1:** Data statistics for SAPO-34 The resolution shells given are the full range, the low-resolution shell and the high-resolution shell. Note that the crystals diffracted beyond the edge of the detector, so that the high-resolution completeness is reduced. This also affects the statistics. Literature cell dimensions for the CHA framework: *a* = *b* = *c* = 9.371 Å, α = β = γ = 94.70° (Wragg *et al.*, 2010 ▸).

Threshold (keV)	25	60
ΔΦ (°)	0.0148	0.0148
Φ (°)	80	75
Δ_detector_ (mm)	306.044	306.044
Space group	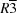	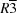
Unit cell (Å, °)		
*a* = *b* = *c*	9.657 (30)	9.609 (2)
α = β = γ	94.210 (41)	94.460 (25)
Resolution (Å)		
Total	9.60–0.65	9.55–0.65
Low-resolution shell	9.60–1.93	9.55–1.92
High-resolution shell	0.69–0.65	0.69–0.65
No. of reflections	3926, 226, 188	3802, 214, 181
No. of unique reflections	1697, 84, 127	1673, 79, 119
Completeness (%)	75.9, 97.7, 34.3	75.3, 91.9, 32.7
*I*/σ(*I*)	2.64, 4.57, 0.95	2.81, 4.89, 1.04
*CC*_1/2_ (%) (Karplus & Diederichs, 2012 ▸)	95.1, 95.3, 48.8	96.3, 95.4, 47.5
*R*_meas_ (%) (Diederichs & Karplus, 1997 ▸)	25.3, 16.8, 53.6	22.9, 17.4, 57.7

**Table 2:** Model statistics for SAPO-34 The literature bond length for P—O = 1.581 (3) Å (Hoppe *et al.*, 1998 ▸) and that for Al—O = 1.761 Å (Jones, 1968 ▸).

Threshold (keV)	25	60
Sum formula	AlPO_4_	AlPO_4_
No. of parameters	56	56
*R*_1_ (%)	25.2	23.7
*R*_complete_ (%) (Luebben & Gruene, 2015 ▸)	25.9	24.3
No. of data [|*F*_o_| 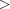 4|σ(*F*_o_)|]	1083	1116
*R*_1_ (%) [|*F*_o_| 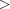 4|σ(*F*_o_)|]	23.4	22.2
*R*_complete_ (%) [|*F*_o_| 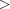 4|σ(*F*_o_)|]	24.2	22.8
〈P—O〉	1.5825 (85)	1.5738 (63)
〈Al—O〉	1.7810 (93)	1.7715 (65)

**Table:** 

*T*	P—O1	P—O2	P—O3	P—O4
25 keV	1.5697 (80)	1.5636 (78)	1.5926 (81)	1.6019 (85)
60 keV	1.5667 (53)	1.5575 (53)	1.5850 (62)	1.5850 (56)

**Table:** 

*T*	Al—O1	Al—O2	Al—O3	Al—O4
25 keV	1.7712 (85)	1.7925 (87)	1.7735 (89)	1.7837 (93)
60 keV	1.7680 (56)	1.7857 (56)	1.7719 (66)	1.7623 (60)
